# The Distinct Roles of LKB1 and AMPK in p53-Dependent Apoptosis Induced by Cisplatin

**DOI:** 10.3390/ijms231710064

**Published:** 2022-09-02

**Authors:** Tatsuya Shimada, Yohsuke Yabuki, Takuya Noguchi, Mei Tsuchida, Ryuto Komatsu, Shuhei Hamano, Mayuka Yamada, Yusuke Ezaki, Yusuke Hirata, Atsushi Matsuzawa

**Affiliations:** Laboratory of Health Chemistry, Graduate School of Pharmaceutical Sciences, Tohoku University, Sendai 980-8578, Japan

**Keywords:** LKB1, AMPKα, apoptosis, cisplatin, DNA damage, DNA damage response

## Abstract

Liver kinase B1 (LKB1) is a serine/threonine protein kinase that acts as a key tumor suppressor protein by activating its downstream kinases, such as AMP-activated protein kinase (AMPK). However, the regulatory actions of LKB1 and AMPK on DNA damage response (DDR) remain to be explored. In this study, we investigated the function of LKB1 in DDR induced by cisplatin, a representative DNA-damaging agent, and found that LKB1 stabilizes and activates p53 through the c-Jun N-terminal kinase (JNK) pathway, which promotes cisplatin-induced apoptosis in human fibrosarcoma cell line HT1080. On the other hand, we found that AMPKα1 and α2 double knockout (DKO) cells showed enhanced stabilization of p53 and increased susceptibility to apoptosis induced by cisplatin, suggesting that AMPK negatively regulates cisplatin-induced apoptosis. Moreover, the additional stabilization of p53 and subsequent apoptosis in AMPK DKO cells were clearly canceled by the treatment with the antioxidants, raising the possibility that AMPK suppresses the p53 activation mediated by oxidative stress. Thus, our findings unexpectedly demonstrate the reciprocal regulation of p53 by LKB1 and AMPK in DDR, which provides insights into the molecular mechanisms of DDR.

## 1. Introduction

Liver kinase B1 (LKB1), also known as serine threonine kinase 11 (STK11), is a tumor suppressor protein originally identified as the causative gene of Peutz-Jeghers syndrome (PJS), which is closely associated with the development of gastrointestinal polyps and cancer [1,2]. In addition, various mutations of LKB1 have been identified in solitary cancers, including breast cancer, lung adenocarcinoma, and cervical cancer [3]. Functionally, LKB1 is activated through the complex formation together with the pseudokinase Ste20-related kinase adaptor protein (STRAD) and the scaffolding protein MO25 [4,5]. When LKB1 is activated by the complex formation, LKB1 phosphorylates and activates at least thirteen substrates [6]. In particular, the AMP-activated protein kinase (AMPK), a kinase complex that consists of a catalytic subunit (AMPKα) and two regulatory subunits (AMPKβ and AMPKγ), is a representative downstream of LKB1 that plays an essential role in the cellular energy homeostasis [7]. The LKB1-AMPK pathway basically exerts tumor suppressive functions by regulating energy metabolism, cell polarity, cell cycle, and apoptosis, and its activation is impaired in patients with various types of cancer [8,9,10]. On the other hand, in some cases, LKB1 contributes to cancer development. For instance, overexpression of LKB1 found in the hepatocellular carcinoma (HCC) is associated with poor prognosis, and LKB1 plays an important role in the survival of circulating tumor cells (CTCs) during the early stages of cell proliferation [11,12]. Moreover, AMPK has also been reported to promote cancer development. AMPK contributes to tumor growth by promoting bioenergetics or activating protein kinase B (Akt) [1,13]. Therefore, further studies are required to develop therapeutic strategies by targeting the LKB1-AMPK axis.

DNA damage response (DDR) is a critical cellular mechanism to prevent the accumulation of DNA damage that causes cancer development through its mutagenic consequences [14,15]. The tumor suppressor p53 is a master regulator of DDR that determines cell survival or death as outcomes of DDR, and therefore, its deletion allows tumorigenesis [14]. Cisplatin, an anti-cancer drug most widely prescribed for cancer therapy, causes DNA damage that leads to the activation of p53, and then the induction of p53-dependent apoptosis in cancer cells [16,17,18]. However, there is a critical problem in that its toxicity sometimes perturbs cellular homeostasis in normal cells, and thereby initiates various side effects [19]. Moreover, cancer cells frequently acquire cisplatin resistance, but its mechanisms are poorly understood [20]. Therefore, studying the cellular responses to cisplatin may provide therapeutic benefit to reduce risk of the adverse reactions and to overcome cisplatin resistance in cancer therapy.

In the present study, we examined the potential role of LKB1 in cisplatin-induced apoptosis and found that LKB1 promotes cisplatin-induced apoptosis through the stabilization of p53. On the other hand, although AMPK acts as a downstream kinase of LKB1, AMPK did not participate in LKB1-mediated p53 stabilization. Rather, AMPK suppressed the p53 stabilization mediated by a mechanism dependent on oxidative stress. Thus, our data demonstrate the opposite roles of LKB1 and AMPK in response to cisplatin, which may help to elucidate the mechanisms underlying cisplatin resistance.

## 2. Results

### 2.1. LKB1 Promotes Cisplatin-Induced p53 Activation and Apoptosis

**Figure 1:**
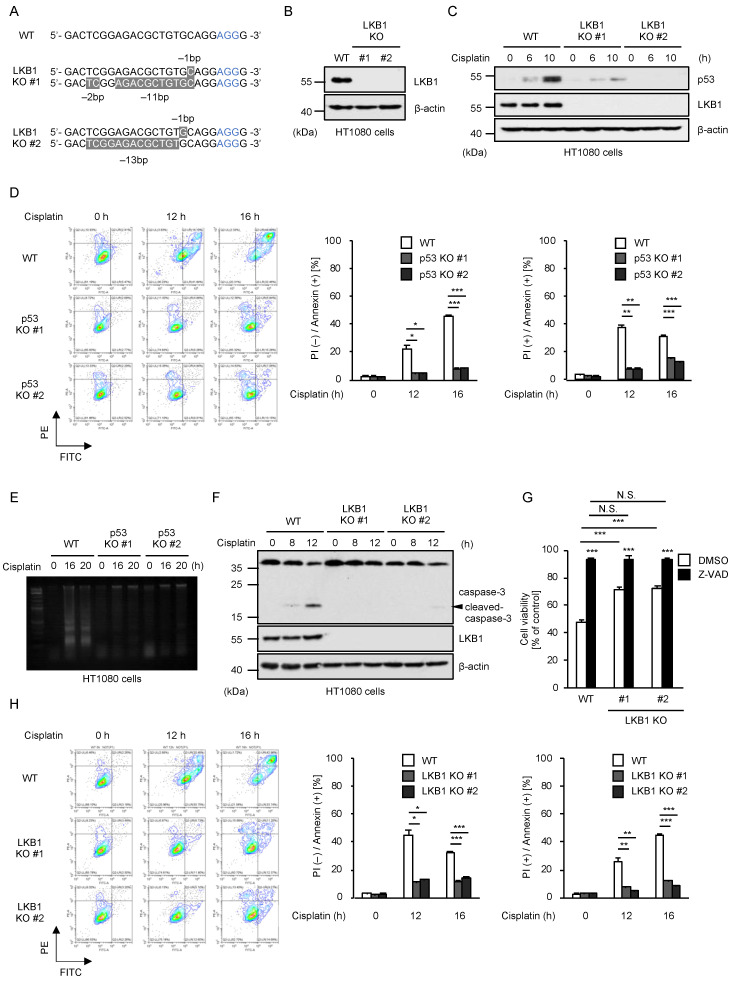
LKB1 promotes cisplatin-induced p53 activation and apoptosis. (**A**) DNA sequences around the guide RNAs (gRNAs) target sites of LKB1. (**B**) Immunoblot analysis of LKB1 in HT1080 cells. Cell lysates were subjected to immunoblotting with the indicated antibodies. β-actin was used as a loading control. (**C**) HT1080 cells were treated with cisplatin (25 μM) for the indicated periods. Cell lysates were subjected to immunoblotting with the indicated antibodies. β-actin was used as a loading control. (**D**) HT1080 cells were treated with cisplatin (25 μM) for the indicated periods. Apoptotic cells were labeled with annexin V-FITC and PI for 15 min and analyzed by FACS. The data were converted to FITC-PE fluorescence density plots. (**E**) HT1080 cells were treated with cisplatin (25 μM) for the indicated periods, and DNA fragmentation was assessed by 2% agarose gel electrophoresis. (**F**) HT1080 cells were treated with cisplatin (25 μM) for the indicated periods. Cell lysates were subjected to immunoblotting with the indicated antibodies. β-actin was used as a loading control. (**G**) HT1080 cells were treated with cisplatin (25 μM) for 24 h in the presence of DMSO or Z-VAD-fmk (20 μM), and then subjected to cell viability assay. Cell viability was measured by PMS (phenazine methosulfate)/MTS (3-(4,5-dimethylthiazol-2-yl)-5-(3-carboxymethoxyphenyl)-2-(4-sulfophenyl)-2H-tetrazolium) assay. (**H**) HT1080 cells were treated with cisplatin (25 μM) for the indicated periods. Apoptotic cells were labeled with annexin V-FITC and PI for 15 min and analyzed by FACS. The data were converted to FITC-PE fluorescence density plots. Data shown are the mean ± SD (n = 3). Significant differences were determined by one-way ANOVA, followed by Tukey–Kramer test; * *p* < 0.05, ** *p* < 0.01, *** *p* < 0.001, N.S.: not significant (vs. control cells). All data are representative of at least three independent experiments.

To explore the potential roles of LKB1 in DDR induced by cisplatin, we established two independent clones of LKB1 knockout cells (LKB1 KO) in human fibrosarcoma cell line HT1080 by using the Clustered Regularly Interspaced Short Palindromic Repeats/CRISPR-associated protein-9 nuclease (CRISPR/Cas9) system as previously described (Figure 1A,B) [21]. In LKB1 KO HT1080 cells, we found that cisplatin-induced p53 stabilization that indicates its activation was clearly attenuated, suggesting that LKB1 positively regulates cisplatin-induced p53 stabilization (Figure 1C). Since we have previously demonstrated that the p53 stabilization triggered by cisplatin contributes to the induction of apoptosis, we expected that LKB1 is required for cisplatin-induced apoptosis [18]. At first, we confirmed the requirement of p53 for cisplatin-induced apoptosis. As shown in Figure 1D, quantitative analysis using fluorescence-activated cell sorting (FACS) after staining with annexin V-FITC and propidium iodide (PI) showed that annexin V-positive (apoptotic) cells were significantly reduced in p53 KO HT1080 cells, when compared with WT HT1080 cells. Moreover, DNA fragmentation associated with apoptosis was suppressed in p53 KO HT1080 cells (Figure 1E). Therefore, these observations show that p53 is required for cisplatin-induced apoptosis in HT1080 cells. It has been well established that an enzymatic function of caspase-3 acts as a main executor of apoptosis, yet recent evidence has shown its non-enzymatic functions [22,23,24,25]. As shown in Figure 1F, cisplatin clearly promoted the processing of caspase-3 into its active form, which was abrogated in LKB1 KO HT1080 cells. Consistent with the caspase-3 activation, cell viability in the presence of cisplatin was recovered in LKB1 KO HT1080 cells (Figure 1G). In addition, the reduction in cell viability was mostly cancelled by co-treatment with Z-VAD-fmk, a pan-caspase inhibitor (Figure 1G). Moreover, the resistance of LKB1 KO HT1080 cells to cisplatin-induced apoptosis was further supported by FACS analysis (Figure 1H). Thus, these results suggest that LKB1 promotes cisplatin-induced apoptosis through the p53 stabilization. 

### 2.2. Kinase Activity of LKB1 Is Required for Cisplatin-Induced p53 Activation and Apoptosis

**Figure 2:**
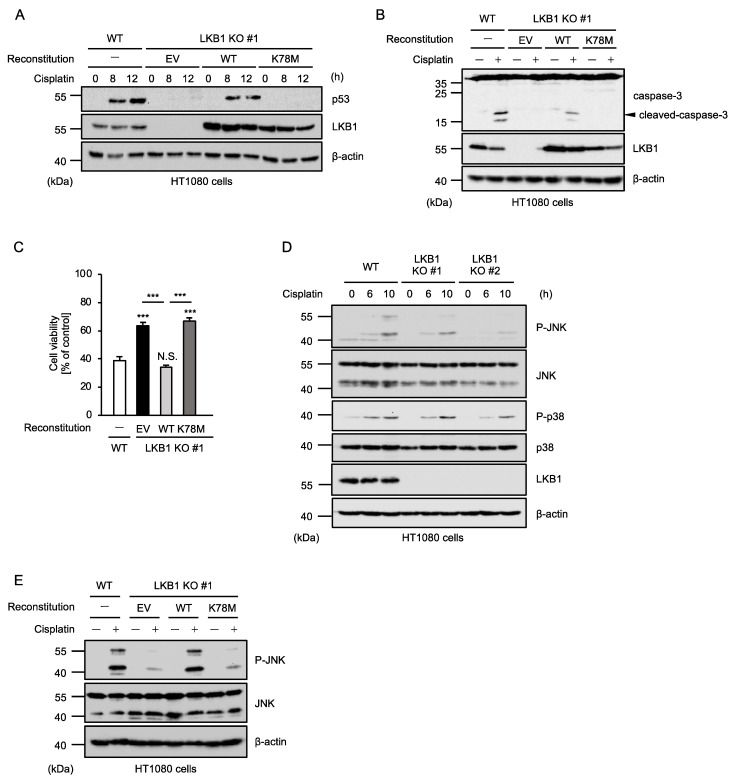
Kinase activity of LKB1 is required for cisplatin-induced p53 activation and apoptosis. (**A**) HT1080 cells were treated with cisplatin (25 μM) for the indicated periods. Cell lysates were subjected to immunoblotting with the indicated antibodies. β-actin was used as a loading control. (**B**) HT1080 cells were treated with cisplatin (25 μM) for 12 h. Cell lysates were subjected to immunoblotting with the indicated antibodies. β-actin was used as a loading control. (**C**) HT1080 cells were treated with cisplatin (25 μM) for 24 h and then subjected to PMS/MTS assay. Data shown are the mean ± SD (n = 3). Significant differences were determined by one-way ANOVA, followed by Tukey–Kramer test; *** *p* < 0.001, N.S.: not significant (vs. control cells). (**D**) HT1080 cells were treated with cisplatin (25 μM) for the indicated periods. Cell lysates were subjected to immunoblotting with the indicated antibodies. β-actin was used as a loading control. (**E**) HT1080 cells were treated with cisplatin (25 μM) for 12 h. Cell lysates were subjected to immunoblotting with the indicated antibodies. β-actin was used as a loading control. All data are representative of at least three independent experiments.

We next examined whether the kinase activity of LKB1 is required for cisplatin-induced apoptosis. To this end, we established LKB1-reconstituted HT1080 cells as previously described (Figure 2A) [26]. The reconstitution of LKB1 wild type (WT) in LKB1 KO HT1080 cells successfully restored the stabilization of p53, whereas that of an enzymatically inactive mutant of LKB1 (K78M mutant) in which lysine (K) 78 was substituted by methionine (M) failed to do so (Figure 2A). Moreover, the reconstitution of LKB1 WT but not the K78M mutant restored cisplatin-induced caspase-3 activation (Figure 2B). Consistent with these observations, cisplatin-induced apoptosis was not sensitized by the reconstitution of the LKB1 K78M mutant (Figure 2C). These results, therefore, suggest that the kinase activity of LKB1 is required for cisplatin-induced apoptosis. Mitogen-activated protein kinases (MAPKs) such as c-Jun N-terminal kinase (JNK) and p38 MAPK have been identified as kinase signaling pathways that positively regulate p53 activation [27,28,29]. In addition, our previous study demonstrated that cisplatin-induced p53 stabilization is mediated by JNK but not p38 MAPK at least in HT1080 cells [18]. We then tested whether LKB1 is involved in the JNK-p53 pathway. As shown in Figure 2D, cisplatin-induced JNK activation (phosphorylation of threonine 183 and tyrosine 185) is partially but certainly attenuated in LKB1 KO HT1080 cells. Furthermore, cisplatin-induced JNK activation was restored by the reconstitution of LKB1 WT but not the K78M mutant (Figure 2E). Therefore, LKB1 appears to positively regulate the p53 stabilization and subsequent apoptosis through the JNK activation.

### 2.3. AMPK Negatively Regulates Cisplatin-Induced p53 Activation and Apoptosis

**Figure 3:**
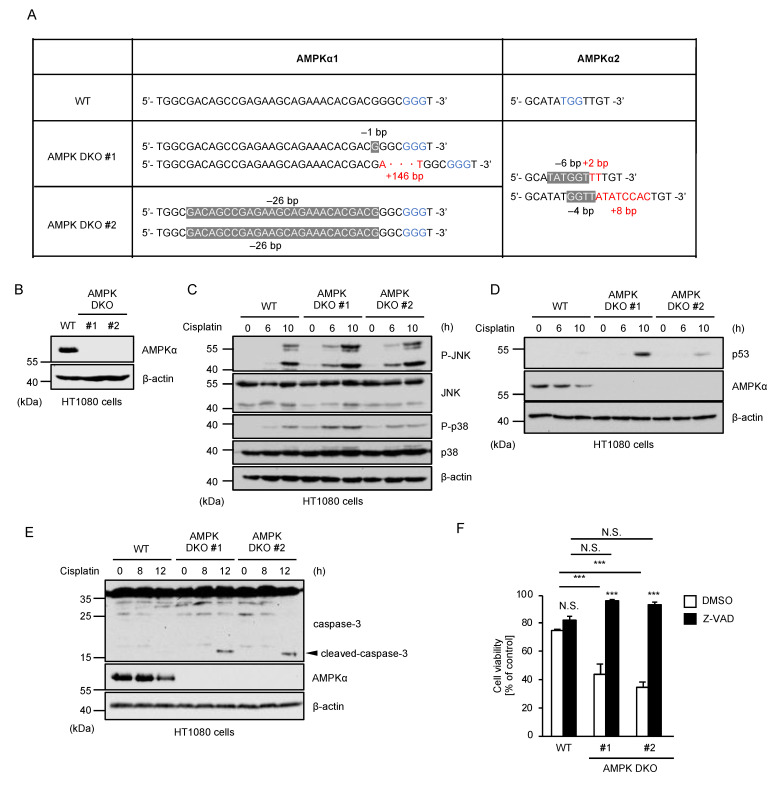

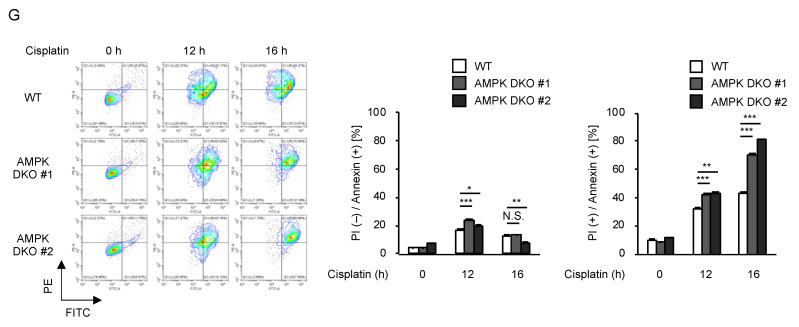
AMPK negatively regulates cisplatin-induced p53 activation and apoptosis. (**A**) DNA sequences around the guide RNAs (gRNAs) target sites of AMPKα1 and AMPKα2. Blue letters: PAM sequences. White letters in the gray markers: sequences deleted by genetic manipulation. Red letters: sequences newly inserted by genetic manipulation. (**B**) Immunoblot analysis of AMPKα in HT1080 cells. Cell lysates were subjected to immunoblotting with the indicated antibodies. β-actin was used as a loading control. (**C**–**E**) HT1080 cells were treated with cisplatin (25 μM) for the indicated periods. Cell lysates were subjected to immunoblotting with the indicated antibodies. β-actin was used as a loading control. (**F**) HT1080 cells were treated with cisplatin (25 μM) for 24 h in the presence of DMSO or Z-VAD-fmk (20 μM), and then subjected to PMS/MTS assay. (**G**) HT1080 cells were treated with cisplatin (20 μM) for the indicated periods. Apoptotic cells were labeled with annexin V-FITC and PI for 15 min and analyzed by FACS. The data were converted to FITC-PE fluorescence density plots. Data shown are the mean ± SD (n = 3). Significant differences were determined by one-way ANOVA, followed by Tukey–Kramer test; * *p* < 0.05, ** *p* < 0.01, *** *p* < 0.001, N.S.: not significant (vs. control cells). All data are representative of at least three independent experiments.

Since AMPK is a representative downstream kinase of LKB1, we next investigated whether AMPK is involved in the LKB1-JNK-p53 pathway. To this end, we established double knockout (DKO) HT1080 cells of AMPKα1 and α2 that function as catalytic subunits of the AMPK kinase complex (Figure 3A,B). Unexpectedly, cisplatin-induced JNK activation was not attenuated, but was rather enhanced in AMPK DKO HT1080 cells (Figure 3C). Moreover, AMPK DKO HT1080 cells showed increased stabilization of p53 and activation of caspase-3 (Figure 3D,E). Consistent with these observations, AMPK DKO HT1080 cells are more sensitive to cisplatin (Figure 3F), and the sensitivity was mostly cancelled by co-treatment with Z-VAD-fmk (Figure 3F). Moreover, FACS analysis revealed that annexin V (+)/PI (+) (late phase apoptosis) cells were significantly increased in AMPK DKO HT1080 cells in the presence of cisplatin, showing that cisplatin-induced apoptosis is accelerated in AMPK DKO HT1080 cells (Figure 3G). Collectively, these observations suggest that AMPK is not involved in the positive regulation of p53 mediated by the LKB1-JNK pathway, and rather negatively regulates cisplatin-induced p53 activation and apoptosis through a distinct mechanism.

### 2.4. AMPK Prevents the Additional Activation of p53 Mediated by Oxidative Stress

**Figure 4:**
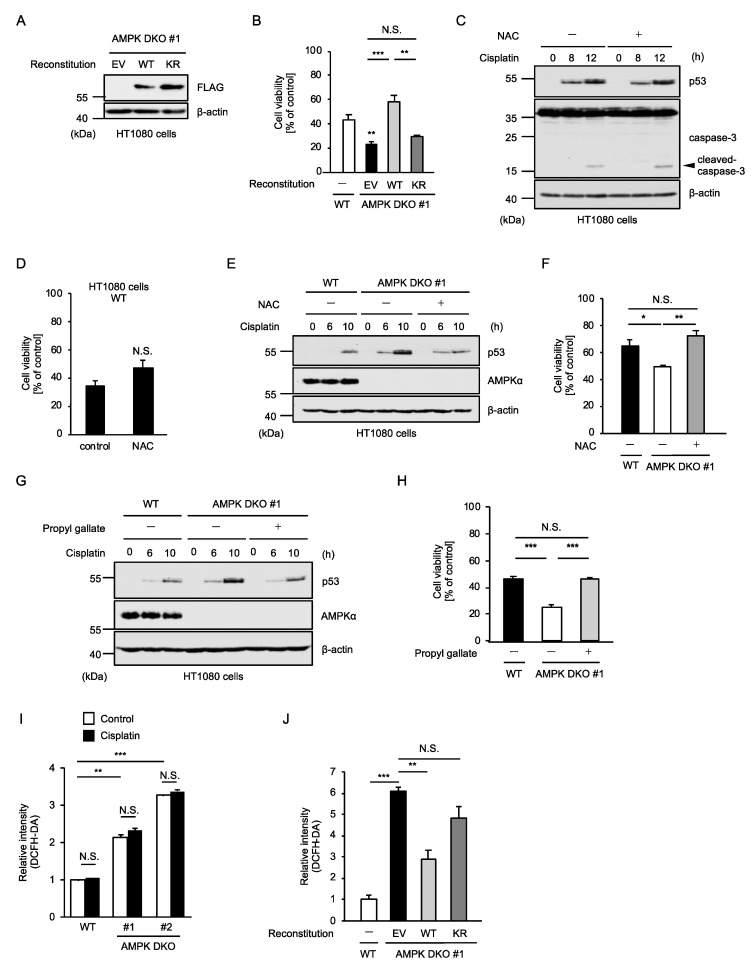
AMPK prevents the additional activation of p53 mediated by oxidative stress. (**A**) Immunoblot analysis of AMPK in HT1080 cells. Cell lysates were subjected to immunoblotting with the indicated antibodies. β-actin was used as a loading control. (**B**) HT1080 cells were treated with cisplatin (25 μM) for 24 h and then subjected to PMS/MTS assay. Data shown are the mean ± SD (n = 3). Statistical significance was tested using an unpaired Student’s t-test; ** *p* < 0.01, *** *p* < 0.001, N.S.: not significant (vs. control cells). (**C**) HT1080 cells were treated with cisplatin (25 μM) for the indicated periods in the presence of DMSO or *N*-acetylcysteine (1 mM). Cell lysates were subjected to immunoblotting with the indicated antibodies. β-actin was used as a loading control. (**D**) HT1080 cells were treated with cisplatin (25 μM) for 24 h in the presence of DMSO or *N*-acetylcysteine (1 mM), and then subjected to PMS/MTS assay. Data shown are the mean ± SD (n = 3). Significant differences were determined by one-way ANOVA, followed by Tukey–Kramer test; N.S.: not significant (vs. control cells). (**E**) HT1080 cells were treated with cisplatin (25 μM) for the indicated periods in the presence of DMSO or *N*-acetylcysteine (1 mM). Cell lysates were subjected to immunoblotting with the indicated antibodies. β-actin was used as a loading control. (**F**) HT1080 cells were treated with cisplatin (25 μM) for 24 h in the presence of DMSO or *N*-acetylcysteine (1 mM), and then subjected to PMS/MTS assay. Data shown are the mean ± SD (n = 3). Significant differences were determined by one-way ANOVA, followed by Tukey–Kramer test; * *p* < 0.01, ** *p* < 0.001, N.S.: not significant (vs. control cells) (**G**) HT1080 cells were treated with cisplatin (25 μM) for the indicated periods in the presence of DMSO or propyl gallate (40 μM). Cell lysates were subjected to immunoblotting with the indicated antibodies. β-actin was used as a loading control. (**H**) HT1080 cells were treated with cisplatin (25 μM) for 24 h in the presence of DMSO or propyl gallate (40 μM), and then subjected to PMS/MTS assay. Data shown are the mean ± SD (n = 3). (**I**) HT1080 cells were treated with cisplatin (25 μM) or saline (control) for 8 h and then incubated with 10 μM 2′,7′-dichlorodihydrofluorescein diacetate (DCFH-DA). Quantification of ROS was calculated by detecting the fluorescence intensity of DCFH-DA. (**J**) HT1080 cells were incubated with 10 μM DCFH-DA. Quantification of ROS was calculated by detecting the fluorescence intensity of DCFH-DA. Significant differences were determined by one-way ANOVA, followed by Tukey–Kramer test; * *p* < 0.05, ** *p* < 0.01, *** *p* < 0.001, N.S.: not significant. (vs. control cells). All data are representative of at least three independent experiments.

We then examined how AMPK suppresses cisplatin-induced p53 activation and apoptosis. Firstly, we established AMPK-reconstituted HT1080 cells, in order to investigate whether the kinase activity of AMPK is required for cisplatin resistance (Figure 4A). The increased sensitivity of AMPK DKO HT1080 cells to cisplatin-induced apoptosis was restored by the reconstitution of AMPKα1 WT but not the kinase dead mutant (K56R mutant) in which lysine (K) 56 was substituted by arginine (R), suggesting that the kinase activity of AMPK is required for the suppression of cisplatin-induced apoptosis (Figure 4B). Accumulating evidence shows that a wide variety of therapeutic agents initiate oxidative stress through reactive oxygen species (ROS) generation, which is thought to be responsible for adverse reactions to these agents [30,31,32,33,34]. In this regard, cisplatin has also been reported to initiate oxidative stress [35,36]. However, cisplatin-induced p53 stabilization and caspase-3 activation were not affected by the treatment with *N*-acetylcysteine (NAC), a representative antioxidant (Figure 4C). Consistent with this observation, NAC failed to protect cells from cisplatin-induced apoptosis, suggesting that oxidative stress is not involved in cytotoxic effects of cisplatin at least in HT1080 cells (Figure 4D). On the other hand, in AMPK DKO HT1080 cells, NAC reduced cisplatin-induced stabilization of p53 to the same extent as WT HT1080 cells (Figure 4E). Interestingly, NAC-treated AMPK DKO HT1080 cells showed similar sensitivity to cisplatin as WT HT1080 cells (Figure 4F). Moreover, the same results were obtained when treated with propyl gallate, a synthetic antioxidant, instead of NAC (Figure 4G,H). Thus, these observations raise the possibility that degree of oxidative stress affects the sensitivity to cisplatin. To address this possibility, we investigated production of ROS by using the ROS indicator 2′, 7′-dichlorodihydrofluorescein diacetate (DCFH-DA). Unexpectedly, the ROS levels were substantially increased in AMPK DKO HT1080 cells under unstimulated conditions (Figure 4I). Moreover, the ROS levels were reduced by the reconstitution of AMPKα1 WT but not the kinase dead mutant, suggesting that the kinase activity of AMPK is required to prevent excessive ROS accumulation (Figure 4J). On the other hand, the ROS levels in both WT and AMPK DKO HT1080 cells were not changed by cisplatin treatment (Figure 4I). Therefore, cisplatin may not cause oxidative stress at least in HT1080 cells. Taken together, these results suggest that the ROS accumulation due to the lack of AMPK somehow promotes cisplatin-induced p53 activation, and thereby promotes cisplatin-induced apoptosis.

## 3. Discussion

**Figure 5:**
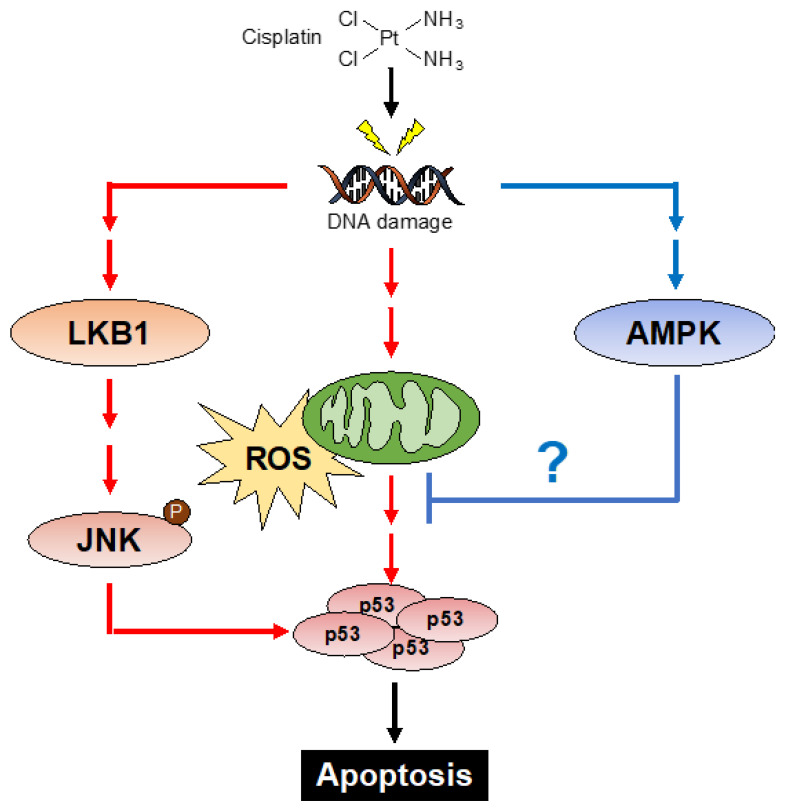
Schematic model to explain our study is described See Section 3.

In the present study, we demonstrate the reciprocal regulation of p53 by LKB1 and AMPK. We found that LKB1 promotes cisplatin-induced p53 activation and subsequent apoptosis. Since it is well known that LKB1 acts as a tumor suppressor protein, the function of LKB1 that positively regulates p53 seems to be reasonable. Mechanistically, our data suggest that LKB1 regulates p53 by promoting cisplatin-induced JNK activation (Figure 5). Although LKB1 has been suggested to be a JNK activator, its mechanism remains unknown [37]. In this regard, our recent study demonstrated that tumor necrosis factor receptor-associated factor 2 (TRAF2), a member of the TRAF family proteins, is involved in the JNK activation in HT1080 cells [18]. In addition, TRAF2 regulates the JNK signaling pathway by activating the upstream kinases of JNK, such as MAPK/ERK kinase kinase-1 (MEKK1) and apoptosis signal-regulating kinase 1 (ASK1) [38,39,40,41]. Therefore, investigating mechanistic links between LKB1 and these proteins that stimulate the JNK signaling pathway may lead to elucidation of the mechanism by which LKB1 regulates the JNK activation.

On the other hand, we found that AMPK is not involved in the LKB1-mediated p53 activation. Furthermore, AMPK appears to negatively regulate the p53 activation through distinct mechanisms. It has been demonstrated that AMPK prevents NADPH oxidase-dependent p53 activation under high-nutrient conditions, which is an example of AMPK as a negative regulator of p53, and a possible mechanism that can explain our observations [42]. On the other hand, under low nutrient conditions, AMPK promotes the p53 activation, and thereby mediates cell cycle arrest [43,44]. These dual functions of AMPK suggest that AMPK tightly controls the activation of p53 positively and negatively, depending on the cellular context and environment.

It was unexpected that AMPK does not conform to the canonical LKB1 pathway, but it was not surprising that AMPK suppresses oxidative-stress-dependent mechanisms associated with the p53 stabilization. Basically, AMPK plays an essential role in cellular survival by maintaining energy homeostasis [7]. In particular, AMPK protects cells from oxidative stress through the induction of antioxidant proteins and autophagy [45,46,47,48]. Moreover, AMPK regulates mitochondrial reactive oxygen species (mtROS), which prevents accumulation of oxidative stress and subsequent senescence [49]. Therefore, it is likely that AMPK regulates cellular redox balance by inducing antioxidant programs in the presence of cisplatin, resulting in the negative regulation of cisplatin-induced apoptosis (Figure 5).

Cisplatin causes DNA damage that initiates p53-dependent apoptosis, resulting in cancer cell death [17,50]. Therefore, cisplatin serves as a chemotherapeutic drug widely used for the treatment of a wide range of solid tumors [51]. However, an important issue is that cancer cells tend to acquire resistance to cisplatin [20]. Although the mechanisms of cisplatin resistance are not fully understood, disruption of the p53 pathway causes cisplatin resistance in cancer cells [52,53]. Since LKB1 KO HT1080 cells exhibited substantial resistance to cisplatin, as shown in Figure 1E, loss-of-function mutations in the LKB1 gene may contribute to the therapeutic resistance to cisplatin. On the other hand, recent studies have demonstrated that AMPK is upregulated or chronically activated in various cancer cells [54,55]. Although the involvement of AMPK in therapeutic resistance to cisplatin remains unclear, our results demonstrated that the loss of AMPK enhanced sensitivity to cisplatin (Figure 3F). This finding raises the possibility that specific inhibitors of AMPK assist the pharmacological effects of cisplatin by enhancing the sensitivity. Thus, our findings demonstrate opposite roles of LKB1 and AMPK in DDR, which may provide new insights into therapeutic strategies to overcome cisplatin resistance.

## 4. Materials and Methods

### 4.1. Cell Culture and Reagents

Human fibrosarcoma cell line HT1080 was grown in Dulbecco’s Modified Eagle Medium (DMEM), 10% heat-inactivated fetal bovine serum (FBS), and 1% penicillin–streptomycin solution, at 37 °C under a 5% CO_2_ atmosphere. All reagents were obtained from commercial sources: cisplatin (CDDP), N-acetylcysteine (NAC), 2′,7′-dichlorodihydrofluorescein diacetate (DCFH-DA) (WAKO, Osaka, Japan), propyl gallate (Sigma, St. Louis, MO, USA), and Z-VAD-fmk (peptide institute, Osaka, Japan). The antibodies used were against FLAG (Sigma), LKB1, total AMPKα, caspase-3 (Santa Cruz, Dallas, USA), phospho-p38 (threonine 180 and tyrosine 182), total p38, phospho-JNK (threonine 183 and tyrosine 185), total JNK, p53 (Cell signaling technology, Danvers, USA), and β-actin (Wako).

### 4.2. PMS/MTS Assay

Cell viability assay was performed as described previously [56]. Cells were seeded on 96-well plates. After indicated stimulation, cell viability was measured by phenazine methosulfate (PMS)/3-(4,5-dimethylthiazol-2-yl)-5-(3-carboxymethoxyphenyl)-2-(4-sulfophenyl)-2H-tetrazolium, inner salt (MTS) assay using a Cell Titer 96 Cell Proliferation Assay kit (Promega, Madison, USA), according to the manufacturer’s protocol. The absorbance was read at 492 nm using a microplate reader. Data are normalized to control (100%) without stimulus.

### 4.3. Immunoblot Analysis

Cells were lysed with DISC lysis buffer TX (20 mM Tris-HCl (pH 7.4), 150 mM NaCl, 1% Triton X-100, 10% Glycerol, and 1% protease inhibitor cocktails (Nacalai Tesque, Kyoto, Japan)). After centrifugation, the cell extracts were resolved by SDS-PAGE and analyzed as described previously [57]. The blots were developed with ECL (Merck Millipore).

### 4.4. Generation of Knockout Cell Lines

All KO cells were generated using the CRISPR/Cas9 system, as described previously [21,58]. Guide RNAs (gRNAs) were designed to target a region in exon 1 of the hLKB1 gene (5′-GACTCGGAGACGCTGTGCAGG-3′), that in exon 1 of the hPRKAA1 (AMPKα1) gene (5′-GAAGCAGAAACACGACGGGC-3′), and that in exon 4 of the hPRKAA2 (AMPKα2) gene (5′-GGATTACTGTCATAGGCATA-3′), using CRISPRdirect (https://crispr.dbcls.jp accessed on 11 July 2017). Then, gRNA-encoding oligonucleotide was cloned into lenti-CRISPRv2 plasmid (addgene, Watertown, MA, USA), and the plasmid was transfected with HEK293A cells together with a packaging plasmid psPAX2 and an envelope plasmid pVSV-G. The supernatants were collected and used to infect HT1080 cells, and then infected cells were selected with puromycin (LKB1 and PRKAA1) or blasticidin (PRKAA2) and cloned by limiting dilution to obtain 100% efficiency. To determine the mutations of hLKB1, hPRKAA1, and hPRKAA2 in cloned cells, the genomic sequence around the target region was analyzed by PCR-direct sequencing using extracted DNA from each clone as a template and the following primers: 5′-GACTGACGTGTAGAACAATC-3′ and 5′-CGCTGCGACAACTGGCCTTG-3′ for hLKB1; 5′-CTTCACTTTGCCGAAGGTGC-3′ and 5′-GGCGGGTACTGGTGATTCTC-3′ for hPRKAA1; 5′-ATGCAGTTTCTTTTGTGCTTGA-3′ and 5′-CATGGTACAGAACGTACAAGGT-3′ for hPRKAA2.

### 4.5. Generation of Reconstituted Sells

Reconstituted HT1080 cells were generated by retroviral transduction as previously described [26]. A packaging cell line Phoenix-AMPHO was transfected with pMXs-IH inserted with LKB1 WT, LKB1 KM mutant, AMPKα1 WT, and AMPKα1 KR mutant. After 48 h, the growth medium containing retrovirus was collected. HT1080 cells were incubated with the virus-containing medium with 10 μg/mL polybrene for 48 h, and uninfected cells were eliminated by hygromycin selection.

### 4.6. FACS Analysis

FACS analysis was performed as described previously [59]. For measurement of ROS generation, HT1080 cells were treated with cisplatin and then incubated with 2′,7′-dichlorodihydrofluorescein diacetate (DCFH-DA) (Wako, Osaka, Japan) for 30 min. The cells were scraped from the culture dishes and dispersed by pipetting as mildly as possible to avoid mechanical damages to the cells. Fluorescence intensity was measured by flow cytometry with the excitation wavelength at 488 nm and the emission wavelength at 580 nm, as previously described. For annexin V and propidium iodide (PI) staining, HT1080 cells were treated with ATP and then labeled with annexin V-FITC (MBL, Tokyo, Japan) and PI for 15 min. Fluorescent cells were detected by CytoFLEX (Beckman Coulter), and apoptotic cells were analyzed by using CytoExpert (Beckman Coulter).

### 4.7. DNA Fragmentation Assay

The DNA fragmentation assay was performed as described previously [60]. Briefly, stimulated cells were collected and suspended in lysis buffer (20 mM Tris-HCl (pH 7.4), 10 mM EDTA, and 0.5% Triton X-100), and the lysates were incubated at room temperature for 10 min, followed by centrifugation at 12,000× *g* for 10 min. The supernatants were incubated with 0.2 mg/mL proteinase K and 0.1 mg/mL RNase A for 1 h at 42 °C, purified with phenol/chloroform extraction and ethanol precipitation, and separated on an agarose gel.

### 4.8. Statistical Analysis

The value was expressed as the mean ± standard deviation (S.D.) using Prism software (GraphPad). All experiments were repeated at least three independent times. Multiple-group comparisons were conducted using the one-way ANOVA analysis of variance followed by the Tukey–Kramer test using Prism software (GraphPad). Data were considered significant when * *p* < 0.05, ** *p* < 0.01, and *** *p* < 0.001.

## Data Availability

The data presented in this study are available in article.
